# New practical scoring system to predict post‐endoscopic retrograde cholangiopancreatography pancreatitis: Development and validation

**DOI:** 10.1002/jgh3.12634

**Published:** 2021-08-12

**Authors:** Koichi Fujita, Shujiro Yazumi, Norimitsu Uza, Akira Kurita, Masanori Asada, Yuzo Kodama, Masashi Goto, Toshiro Katayama, Takahiro Anami, Akihiko Watanabe, Atsushi Sugahara, Hidekazu Mukai, Takashi Kawamura

**Affiliations:** ^1^ Department of Gastroenterology and Hepatology Yodogawa Christian Hospital Osaka Japan; ^2^ Department of Preventive Services Kyoto University School of Public Health Kyoto Japan; ^3^ First Research Department Tazuke Kofukai Medical Research Institute Osaka Japan; ^4^ Department of Gastroenterology and Hepatology Kitano Hospital Osaka Japan; ^5^ Department of Gastroenterology and Hepatology Kyoto University Graduate School of Medicine Kyoto Japan; ^6^ Department of Gastroenterology and Hepatology Japanese Red Cross Osaka Hospital Osaka Japan; ^7^ Department of Gastroenterology Kobe University Graduate School of Medicine Kobe Japan; ^8^ Kyoto University Health Service Kyoto Japan; ^9^ Division of General Medicine National Hospital Organization Kyoto Medical Center Kyoto Japan; ^10^ Department of Medical Engineering, Faculty of Health Sciences Morinomiya University of Medical Science Osaka Japan; ^11^ Help Center of Medical Research Tazuke Kofukai Medical Research Institute Osaka Japan; ^12^ Department of Internal Medicine Senriyama Hospital Osaka Japan

**Keywords:** algorithms, endoscopic retrograde cholangiopancreatography, pancreatitis

## Abstract

**Background and Aim:**

Post‐endoscopic retrograde cholangiopancreatography (ERCP) pancreatitis (PEP) is the most serious adverse event of ERCP. Therefore, it is important to identify high‐risk patients who require prophylactic measures. The aim of this study was to develop a practical prediction model for PEP that identifies high‐risk patients.

**Methods:**

Patients who underwent ERCP at three tertiary hospitals between April 2010 and September 2012 were enrolled. The dataset was divided into a training set (two centers) and validation set (one center). Using the training set, univariable and multivariable analyses were performed to identify predictive factors for PEP. We constructed a scoring system and evaluated reproducibility using the validation set.

**Results:**

A total of 2719 ERCPs were analyzed. PEP developed in 133 cases (4.9%). Risk factors (adjusted odds ratios [OR]) in the training set were a history of PEP (OR: 4.2), intact papilla (OR: 2.4), difficult cannulation (OR: 1.9), pancreatic guidewire‐assisted biliary cannulation (OR: 2.2), pancreatic injection (OR: 2.1), pancreatic intraductal ultrasonography (IDUS)/sampling from the pancreatic duct (OR: 2.2), and biliary IDUS/sampling from the biliary tract (OR: 2.8). A scoring system was constructed using these seven clinical variables. The areas under the receiver‐operating characteristic curve of this model were 0.799 in the training set and 0.791 in the validation set. In the high‐risk group at a score of 3 or higher, the incidence of PEP was 13.4%, and all severe/fatal cases were in the high‐risk group.

**Conclusions:**

This scoring system helps to predict each patient's risk and select preventive measures.

## Graphical abstract

We develop and validate a practical prediction model for post‐endoscopic retrograde cholangiopancreatography (ERCP) pancreatitis based on the ERCP database of three tertiary hospitals. This scoring system, which considers the respective weight of seven predictive factors, showed excellent discriminability and reproducibility, and identified high‐risk patients. The accurate quantification of each patient's risk of PEP will reduce unnecessary medication and facilitate the selection of appropriate prophylactic measures by clinicians.

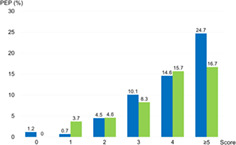

## Introduction

Endoscopic retrograde cholangiopancreatography (ERCP) is an invaluable procedure for the diagnosis and management of pancreaticobiliary diseases. However, it occasionally causes adverse events such as pancreatitis, bleeding, perforation, and infection. Acute pancreatitis is the most common adverse event of ERCP. Post‐ERCP pancreatitis (PEP) may be severe or fatal. The reported incidence of PEP widely varies between 1.6 and 15%, and a systematic survey reported that the cumulative incidence of PEP was 3.47% and case fatality was 0.11%.^1^ Therefore, prophylaxis for PEP is a clinically important issue.

Several strategies have been proposed to prevent PEP, including appropriate patient selection, the rectal administration of nonsteroidal anti‐inflammatory drugs (NSAIDs), and aggressive hydration with lactate Ringer's solution.^2^, ^3^, ^4^, ^5^, ^6^ Pancreatic stenting is considered the most promising prophylactic procedure for high‐risk patients because it maintains the outflow of pancreatic juice.^7^ However, attempting prophylactic pancreatic stenting (PPS) has the potential risk of pancreatitis, pancreatic ductal damage, and inward migration.^8^ Since prophylactic measures sometimes lead to adverse events, they are regarded as a double‐edged sword. To safely perform ERCP, it is important to individually estimate the risk of PEP in each patient and identify high‐risk patients.

Over the past few decades, risk factors for PEP have been the focus of many studies; however, there are currently only a few reliable methods for predicting PEP in individual patients.^6^, ^9^, ^10^

Friedland et al. were the first to propose a scoring system to predict post‐ERCP pancreatitis.^11^ This scoring system consisted of pain during the procedure, pancreatic duct cannulation, a history of PEP, and the cannulation attempt score. However, since pain during the procedure is difficult to measure, a prediction model for PEP needs to consist of more objective and measurable components.

Three more prediction models have recently been reported. However, these models are not suitable for use in clinical practice because of their low discriminability, complexity, or lack of external validation.^12^, ^13^, ^14^

In the present study, we aimed to develop and validate a practical prediction model for PEP based on the ERCP database of three major tertiary hospitals in the western metropolitan area of Japan.

## Methods

### Setting/participants

In our retrospective cohort study, 2976 consecutive ERCP procedures performed from April 2010 and September 2012 were extracted from the databases of Kyoto University Hospital (Kyoto), Kitano Hospital (Osaka), and Yodogawa Christian Hospital (Osaka).

Among these procedures, those with transpapillary ERCP were included in the analysis, whereas those on patients younger than 18 years, with comorbid acute pancreatitis, and with altered gastrointestinal anatomy, such as the Roux‐en‐Y reconstruction, were excluded. All ERCP patients stayed in the hospital for at least 24 h after the procedure to monitor the clinical manifestations of pancreatitis, and received continuous intravenous fluid infusion from around the time of ERCP until the following morning.

### Outcomes

The main outcome was incident PEP. The diagnosis of PEP was based on the consensus definition: new or worsened abdominal pain, new or prolonged hospitalization for at least 2 days, or an increased post‐procedure (>24 h) serum amylase level three‐fold the upper normal limit or higher.^15^, ^16^, ^17^ The severity of PEP was defined by modified Cotton's criteria: mild PEP was defined as a 2–3‐day extension of the hospital stay or fasting; moderate PEP as a 4–10‐day extension of the hospital stay or fasting; and severe PEP as a > 10‐day extension of the hospital stay or fasting, hemorrhagic pancreatitis, pancreatic necrosis, pseudocyst, or the need for percutaneous drainage or surgical intervention. Other outcomes included the adverse events defined in the 2010 American Society for Gastrointestinal Endoscopy (ASGE) lexicon for endoscopic adverse events.^18^

### Predictors

We assessed two types of candidate predictive factors. Patient‐related factors included younger age, female sex, history of PEP, the absence of chronic pancreatitis, normal serum bilirubin, hyperamylasemia before ERCP, periampullary diverticulum, intact papilla, and a suspected sphincter of Oddi dysfunction. Procedure‐related factors included difficult cannulation, pancreatic guidewire (PGW)‐assisted biliary cannulation, precut sphincterotomy, biliary sphincterotomy, biliary balloon sphincter dilation, pancreatic injection, PPS, pancreatic drainage, endoscopic biliary stenting, endoscopic naso‐biliary drainage, endoscopic metallic stenting, the extraction of biliary stones, biliary intraductal ultrasonography (IDUS), pancreatic IDUS, and sampling from the biliary tract and pancreatic duct. In the present study, younger age was defined as <60 years, chronic pancreatitis as the presence of pancreatic stones, and intact papilla as no previous sphincterotomy or papillary balloon dilation or stenting. Difficult cannulation was defined as that taking more than 15 min. Pancreatic drainage included pancreatic stenting and naso‐pancreatic drainage, except for PPS. Sampling was obtained by brushing cytology or forceps biopsy.

### Statistical methods

Categorical data are presented as numbers (percentage) and continuous data as means (standard deviation) for normally distributed data and medians (range) for skewed numerical data.

The dataset was divided into a training set and validation set. The training set used data from two centers, Kyoto University and Yodogawa Christian Hospital, and the validation set used data from one center, Kitano Hospital.

To develop the prediction model, we assessed the multicollinearity of the predictor variables and selected representatives. A univariable analysis was then performed using the chi‐squared test for each of the potential predictors. Candidate predictors were selected based on *P* values <0.2 in the chi‐squared test other than known definite risk factors. A multiple logistic regression analysis was then performed using backward stepwise methods and the odds ratio (OR) and its 95% confidence interval (CI) were indicated.

We constructed a scoring system to predict PEP based on the findings of the multivariable analysis. To generate a simple integer‐based point score for each predictor variable, scores were given by multiplying the β coefficient by 10 and rounding up or down to the nearest integer. The overall risk score for each patient was calculated by summing the scores of all components.

To assess the calibration of the scoring system, the incidence of PEP was plotted against the total score, and a visual inspection of the histogram and the Hosmer–Lemeshow goodness‐of‐fit test were performed. To assess the discrimination of the scoring system, we drew a receiver‐operating characteristic (ROC) curve for the risk of PEP and calculated the area under the ROC curve.

Overfitting and optimism in the training set were evaluated using the bootstrap method by sampling with replacements for 2000 iterations. The area under the ROC curve was calculated in each resampling. Optimism was calculated as the difference between training performance and bootstrap performance.^19^, ^20^

We also evaluated the calibration and discrimination of the validation set and considered risk stratification based on the score–incidence graph and ROC curve in all cases.

Analyses were performed using R 4.0.3 (The R Foundation for Statistical Computing Platform, Vienna, Austria) and JMP10 (SAS Institute, Cary, NC, USA).

### Ethics

The present study was approved by the Institutional Review Boards of Kyoto University, Yodogawa Christian Hospital, and Kitano Hospital. This study was registered in the University Hospital Medical Information Network (UMIN000038243).

## Results

### Patient characteristics

During the study period, 2976 ERCPs were performed at the three participating hospitals. Among these, 257 ERCPs were excluded for the following reasons: patient age <18 years, 10; comorbid acute pancreatitis, 70; altered gastrointestinal anatomy, 114; anastomosis or fistula, 32; data missing, 19, and others, 12. Therefore, 2719 ERCPs were included in the present study. In the patient cohort, 40% were women, and the median age was 67.2 years. ERCP for biliary diseases and that for pancreatic diseases were 73.2 and 26.8%, respectively. The success rate of selective cannulation was 98.5% for all patients and 96.5% for those with intact papilla. The training set consisted of 1969 cases in two centers, while the validation set consisted of 750 cases in one center, and their characteristics were similar (Table 1).

**Table 1:** Baseline characteristics of study participants

	Training set	Validation set	Total
ERCPs, *n*	1969	750	2719
Sex (male/female)	1186/783	446/304	1632/1087
Age, average (range)	66.4 (20–98)	69.3 (21–100)	67.2 (20–100)
Indication			
Biliary disease, *n* (%)	1443 (73.3)	546 (72.8)	1989 (73.2)
Non‐neoplastic/neoplastic	874/569	331/215	1205/784
Pancreatic disease, *n* (%)	526 (26.7)	204 (27.2)	730 (26.8)
Non‐neoplastic/neoplastic	213/313	91/113	304/426
Intact papilla, *n* (%)	883 (44.9)	319 (42.5)	1202 (44.2)
Difficult cannulation, *n* (%)	316 (16.1)	151 (20.1)	476 (17.5)
Selective cannulation, *n* (%)	1939 (98.5)	738 (98.4)	2677 (98.5)
PEP, *n* (%)	96 (4.9)	37 (4.9)	133 (4.9)

ERCP, endoscopic retrograde cholangiopancreatography; PEP, post‐ERCP pancreatitis.

A total of 192 adverse events were documented among 2719 ERCPs (Table 2). PEP developed in 133 cases (4.9%); 80 mild cases, 46 moderate cases, 6 severe cases, and 1 fatal case. Bleeding was detected in 24 cases (0.9%), perforation in 16 cases (0.6%), infection (cholangitis/cholecystitis) in 7 cases (0.3%), and others (including stent migration and respiratory disorders) in 12 cases (0.4%).

**Table 2:** Incidence and severity of adverse events of endoscopic retrograde cholangiopancreatography

		Pancreatitis	Bleeding	Perforation	Infection	Other
Total		133 (4.9%)	24 (0.9%)	16 (0.6%)	7 (0.3%)	12 (0.4%)
Severity	Mild	80	1	6	2	2
	Moderate	46	21	7	5	10
	Severe	6	2	3	0	0
	Fatal	1	0	0	0	0

### Predictor selection

Biliary IDUS and sampling from the biliary tract had multicollinearity for PEP, and their frequencies were low. Therefore, these factors were combined as biliary IDUS and/or sampling from the biliary tract. Similarly, pancreatic IDUS and tissue sampling from the pancreatic duct were combined as pancreatic IDUS and/or sampling from the pancreatic duct. Thirteen candidate factors with *P* values <0.2 were selected in the univariable analysis of the training set: female sex, a history of PEP, the absence of chronic pancreatitis, intact papilla, difficult cannulation, PGW‐assisted biliary cannulation, precut sphincterotomy, biliary sphincterotomy, pancreatic injection, PPS, pancreatic IDUS and/or sampling from the pancreatic duct, the extraction of biliary stones, and biliary IDUS and/or sampling from the biliary tract (Table 3).

**Table 3:** Candidate predictors in a univariable analysis using the training set

Factors	*n*	PEP (%)	OR	95% CI	*P* value
Patient‐related factors					
Younger age	476	27 (5.7)	1.24	0.79–1.96	0.354
Female sex	783	49 (6.3)	1.62	1.07–2.44	0.0206
History of post‐ERCP pancreatitis	109	10 (9.2)	2.08	1.05–4.14	0.032
Absence of chronic pancreatitis	1796	91(5.1)	1.79	0.72–4.47	0.2042
Suspected sphincter of Oddi dysfunction	9	1 (11.1)	2.45	0.30–19.82	0.384
Periampullary diverticulum	331	19 (5.7)	1.23	0.74–2.07	0.4232
Intact papilla	883	74 (8.4)	4.42	2.72–7.18	<0.0001
Procedure‐related factors					
Difficult cannulation	316	43 (13.6)	4.75	3.12–7.25	<0.0001
PGW‐assisted biliary cannulation	136	26 (19.1)	5.95	3.65–9.71	<0.0001
Precut sphincterotomy	39	5 (12.8)	2.97	1.14–7.78	0.02
Biliary sphincterotomy	329	25 (7.6)	1.82	1.13–2.91	0.012
Biliary balloon sphincter dilation	34	3 (8.8)	1.92	0.58–6.38	0.2809
Pancreatic injection	822	72 (8.8)	4.49	2.80–7.20	<0.0001
Pancreatic drainage	242	16 (6.6)	1.46	0.84–2.54	0.1806
Prophylactic pancreatic stent	58	8 (13.8)	3.31	1.52–7.20	0.0014
Pancreatic IDUS/sampling from the pancreatic duct	77	11 (14.3)	3.54	1.81–6.95	<0.0001
Endoscopic biliary stenting	593	24 (4.1)	0.76	0.48–1.23	0.2625
Endoscopic nasobiliary drainage	568	33 (5.8)	1.31	0.85–2.02	0.2203
Self‐expandable metallic stent	100	5 (5.0)	1.03	0.41–2.59	0.9527
Extraction of biliary stones	356	11 (3.1)	0.57	0.30–1.09	0.0839
Biliary IDUS/sampling from the biliary tract	281	33 (11.7)	3.43	2.21–5.34	<0.0001

CI, confidence interval; IDUS, intraductal ultrasonography; OR, odds ratio; PEP, post‐ERCP pancreatitis; PGW, pancreatic guidewire.

The multivariable logistic regression analysis of the training set identified seven significant independent risk factors: two patient‐related and five procedure‐related factors (Table 4). Significant risk factors and their adjusted OR were a history of PEP (OR: 4.2 [95% CI: 1.8–8.8]), intact papilla (OR: 2.4 [95% CI: 1.4–4.5]), difficult cannulation (OR: 1.9 [95% CI: 1.1–3.3]), PGW‐assisted biliary cannulation (OR: 2.2 [95% CI: 1.2–4.0]), pancreatic injection (OR: 2.1 [95% CI:1.2–3.7]), pancreatic IDUS and/or sampling from the pancreatic duct (OR: 2.2 [95% CI: 1.0–4.6]), and biliary IDUS and/or sampling from the biliary tract(OR: 2.8 [95% CI: 1.8–4.5]).

**Table 4:** Predictors in multivariable analysis and their scores

Predictive factors	OR	95% CI	*P* value	β	Score
Patient‐related factors					
History of post‐ERCP pancreatitis	4.2	1.8–8.8	0.0011	0.714	2
Intact papilla	2.4	1.4–4.5	0.0026	0.447	1
Procedure‐related factors					
Difficult cannulation	1.9	1.1–3.3	0.0236	0.321	1
PGW‐assisted biliary cannulation	2.2	1.2–4.0	0.0125	0.391	1
Pancreatic injection	2.1	1.2–3.7	0.0112	0.363	1
Pancreatic IDUS/sampling from the pancreatic duct	2.2	1.0–4.6	0.0447	0.403	1
Biliary IDUS/sampling from the biliary duct	2.8	1.8–4.5	<0.0001	0.521	2

CI, confidence interval; ERCP denotes endoscopic retrograde cholangiopancreatography; IDUS, intraductal ultrasonography; OR, odds ratio; PGW, pancreatic guidewire.

### Construction of the scoring system

The probability of pancreatitis was predicted using the following equation:

$$1/\left(1+e^{1.48}\times e^{-\left[0.71\times \text{history of}\;\mathrm{PEP}+0.45\times \text{intact papilla}+0.32\times \text{difficult cannulation}+0.39\times \mathrm{PGW}-\text{assisted biliary cannulation}+0.36\times \text{pancreatic injection}+0.40\times \text{pancreatic}-\text{IDUS}/\text{sampling from the pancreatic duct}+0.52\times \text{biliary}-\text{IDUS}/\text{sampling from the biliary tract}\right]}\;\right)$$

$$\approx 1/\left(1+e^{1.48}\times e^{-0.34\;\left[2\times \text{history of}\;\mathrm{PEP}+1\times \text{intact papilla}+1\times \text{difficult cannulation}+1\times \mathrm{PGW}-\text{assisted biliary cannulation}+1\times \text{pancreatic injection}+1\times \text{pancreatic}-\text{IDUS}/\text{sampling from the pancreatic duct}+2\times \text{biliary}-\text{IDUS}/\text{sampling from the biliary tract}\right]}\right)$$

$$=1/\left(1+e^{1.48}\times e^{-0.34\times \text{total score}}\right)$$

A simple scoring system was constructed from these clinical variables: a history of PEP (2 points), intact papilla (1 point), difficult cannulation (1 point), PGW‐assisted biliary cannulation (1 point), pancreatic injection (1 point), pancreatic IDUS/sampling from the pancreatic duct (1 point), and biliary IDUS/sampling from the biliary tract (2 points) (Table 4).

### Performance of the scoring system

**Figure 1:**
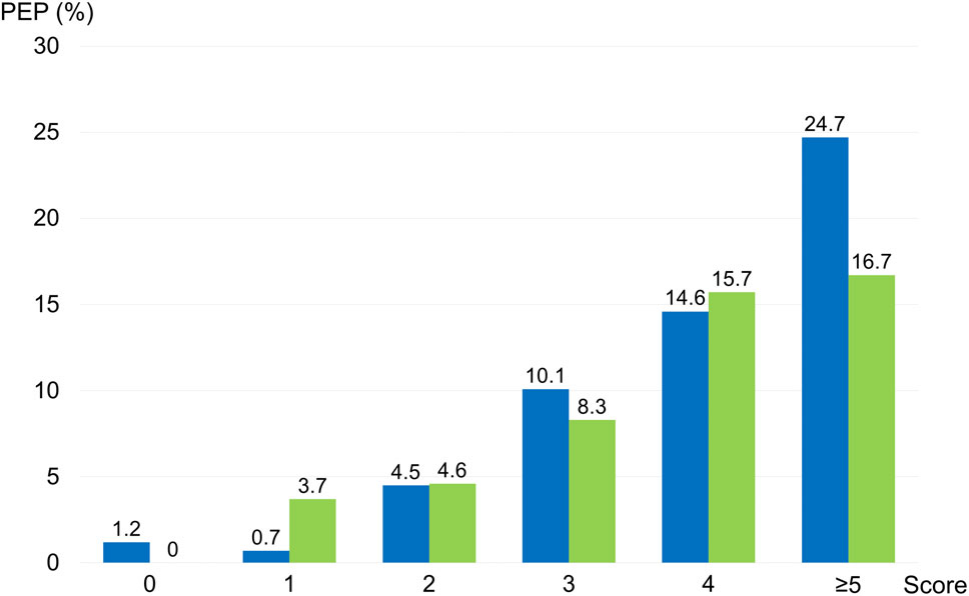
Incidence of post‐ERCP pancreatitis according to the total score in the training set and validation set. 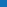, Training set; 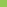, validation set.

In the training set, the incidence of PEP was 1.2% at score 0 (*n* = 661), 0.7% at score 1 (*n* = 426), 4.5% at score 2 (*n* = 399), 10.1% at score 3 (*n* = 248), 13.6% at score 4 (*n* = 158), and 24.7% at score 5 or higher (*n* = 77). The incidence of PEP tended to increase according to the total score (Fig. 1). The *P* value of the Hosmer–Lemeshow goodness‐of‐fit test on the training set was 0.3658, indicating an acceptable fit. The area under the ROC curve of this model was 0.799.

### Internal validation

A bootstrap analysis (i.e. resampling the model 2000 times) revealed a mean over the optimism value of 0.011 (95% CI: −0.034–0.054) and a corrected AUC of 0.788.

### External validation

In the validation set, the incidence of PEP was 0% at score 0 (*n* = 252), 3.7% at score 1 (*n* = 163), 4.6% at score 2 (*n* = 132), 8.3% at score 3 (*n* = 97), 15.7% at score 4 (*n* = 70), and 16.7% at score 5 or higher (*n* = 36). The score–incidence graph of the validation set was similar to that of the training set (Fig. 1). The *P* value of the Hosmer–Lemeshow goodness‐of‐fit test on the validation set was 0.7814. The area under the ROC curve on the validation set was 0.791, which was similar to performance in the training set (0.799).

### Risk stratification

Based on the score–incidence graph and ROC curve, all cases were stratified into two groups: a low‐risk group (scoring 2 points or less) and a high‐risk group (more than 3 points). The incidence of PEP was 2.0% at scores of 0–2 (*n* = 2033), and 13.4% at a score of 3 or higher (*n* = 686). All cases with severe or fatal PEP were classified as the high‐risk group (Table 5).

**Table 5:** Risk stratification by scores

Risk group	Score	*n*	PEP, *n* (%)	Severe/fatal, *n*
Low risk	0–2	2033	41 (2.0)	0
High risk	3–7	686	92 (13.4)	7

PEP, post‐ERCP pancreatitis.

Among the 1381 ERCPs performed on patients without intact papilla and history of PEP, 96.2% had a score of 2 or less (low risk). Among these ERCPs, 19 cases developed PEP, with an incidence of 1.4%.

## Discussion

Our new scoring system, which considers the respective weight of seven predictive factors, indicated excellent discriminability and reproducibility. This scoring system consisted of a history of PEP, intact papilla, difficult cannulation, PGW‐assisted biliary cannulation, pancreatic injection, pancreatic IDUS/sampling from the pancreatic duct, and biliary IDUS/sampling from the biliary tract, which are well‐known risk factors.^21^, ^22^

PEP developed in 13.4% of cases with a score of 3 or higher, and all severe or fatal PEP cases were classified with a score of 3 or higher. Therefore, a score of 3 or higher needs to be considered as high risk and proactive prophylactic measures need to be taken to prevent PEP. According to the 2020 European Society of Gastrointestinal Endoscopy (ESGE) recommendations, 100 mg of diclofenac or indomethacin is administered immediately before ERCP to all patients without contraindications.^6^ However, prior to ERCP, it is possible to determine that most ERCPs for patients without intact papilla and history of PEP are low risk. Since NSAIDs sometimes cause adverse events, such as hypersensitivity reactions, these patients do not require NSAIDs before ERCP; NSAIDs only need to be administered after ERCP if the score reaches 3 or higher.^23^, ^24^

ASGE and ESGE recommend PPS for high‐risk patients with easy pancreatic stenting: PGW‐assisted biliary cannulation, transpancreatic sphincterotomy, and repeated inadvertent main pancreatic duct cannulation.^6^, ^10^ We also recommend PPS for patients with a score of 3 or higher and easy pancreatic stenting, particularly those with PGW‐assisted attempts at biliary cannulation.^25^ Among patients receiving PGW‐assisted biliary cannulation, the incidence of PEP was reported to be significantly lower in those with PPS than in those without stenting (2.9% *vs* 23%).^26^

The effectiveness of aggressive hydration with lactated Ringer's solution was recently demonstrated.^3^, ^4^, ^27^, ^28^ In patients not at risk of fluid overload, it may be useful to initiate aggressive hydration when the score reaches 3 or higher.

Moreover, hospitalization, post‐ERCP blood tests, fasting, and other types of post‐ERCP management need to be provided based on the risk level.

The scoring system of Friedland *et al*. consisted of pain during the procedure, pancreatic duct cannulation, a history of PEP, and the cannulation attempt score.^11^ The most prominent difference between the previous scoring system and ours is objectivity. “Pain during the procedure” and “the cannulation attempt score” are difficult to measure objectively because “pain during the procedure” is markedly affected by sedation levels, the types of analgesic agents used, and patient characteristics, and the definition of “cannulation attempts” is also ambiguous. In addition, our system uniquely included “intact papilla”, which may markedly affect the difficulty of ERCP, and “PGW‐assisted biliary cannulation,” which is closely related to PPS. All of our factors are easy to measure, and, thus, our scoring system may be more reliable in clinical practice.

Chiba *et al*. proposed a prediction model using a propensity score analysis.^14^ Their model consisted of five factors: intact papilla, PGW‐assisted biliary cannulation, difficult cannulation, pancreatic injection, and the absence of a pancreatic stent, four of which are also examined in our model. Therefore, intact papilla, PGW‐assisted biliary cannulation, difficult cannulation, and pancreatic injection are regarded as key factors for predicting PEP. Their model uses a propensity score to estimate the risk of PEP, which needs to be confirmed against a complex look‐up table, whereas our model uses a simple addition of integer scores, which can be easily used by clinicians in the endoscopy room.

The present study had several limitations. NSAIDs were not evaluated because prophylactic NSAIDs had not been used at the three hospitals during the study period. In Japan, the national health insurance system only covers up to 50 mg of diclofenac or indomethacin, and the effectiveness of low‐dose NSAIDs has not been demonstrated. Another limitation is that the present study was ERCP‐based, not patient‐based. Repeated ERCPs were treated as an independent procedure, and this may have induced clustering effects. However, the clinical courses of first and second ERCPs were not necessarily similar, even in the same patient. Therefore, we included all ERCPs.

In conclusion, this scoring system will serve as a useful prediction tool for PEP in clinical practice. For high‐risk patients with a score of 3 or higher, we recommend aggressive preventive measures and close monitoring after ERCP.
